# Counting everyone: evidence for inclusive measures of disability in federal surveys

**DOI:** 10.1093/haschl/qxae106

**Published:** 2024-08-21

**Authors:** Jean P Hall, Kelsey S Goddard, Catherine Ipsen, Andrew Myers, Noelle K Kurth

**Affiliations:** Institute for Health and Disability Policy Studies, University of Kansas, Lawrence, KS 66045, United States; Institute for Health and Disability Policy Studies, University of Kansas, Lawrence, KS 66045, United States; Rural Institute for Inclusive Communities, University of Montana, Missoula, MT 59812, United States; Rural Institute for Inclusive Communities, University of Montana, Missoula, MT 59812, United States; Institute for Health and Disability Policy Studies, University of Kansas, Lawrence, KS 66045, United States

**Keywords:** disability, census, ACS-6, disability measurement

## Abstract

The US Census Bureau has used the American Community Survey six-question set (ACS-6) to identify disabled people since 2008. In late 2023, the Census Bureau proposed changes to these questions that would have reduced disability prevalence estimates by 42%. Because these estimates inform funding and programs that support the health and independence of people with disabilities, many disability researchers and advocates feared this change in data collection would lead to reductions in funding and services. While the Census has paused—but not ruled out—the proposed changes, it is critical that alternate, more inclusive disability questions be identified and tested. We used data from the 2023/2024 National Survey on Health and Disability to explore alternative questions to identify disabled people in national surveys. A single broad question about conditions identified 11.2% more people with disabilities, and missed significantly fewer people with psychiatric disabilities compared to the current ACS-6 questions. A combination of a broad question and the existing ACS-6 questions may be necessary to more accurately and inclusively identify people with disabilities.

## Teaser

A single broad survey question captured more people with disabilities, particularly those with psychiatric disabilities, than the six-question set currently used in the American Community Survey. More inclusive counts of disabled Americans are needed to fully understand and address health disparities for this population.

## Background

In September 2023, the US Census Bureau proposed changes to the six-question set used to identify disabled people in the American Community Survey (known as the ACS-6).^1^ Testing of the replacement questions by the Census Bureau indicated their use would have decreased the prevalence of disability in ACS estimates by approximately 42%.^1,2^ Because these estimates are used to inform funding for programs and services, many disability researchers and advocates feared the proposed change would lead to reduced funding.^3^ While the changes have been paused in response to strong opposition, the situation highlights the growing recognition that existing disability population measures need improvement.^4^ Indeed, research by our team and others demonstrates that the existing ACS-6 questions undercount people with certain disabilities by up to 40%.^5,6^

One issue with both the current ACS-6 questions and the proposed replacement questions is that they ask about functional difficulties rather than directly about disability.^5-7^ The Affordable Care Act, however, specifies that disability be captured as a demographic characteristic in federal surveys to better understand health disparities.^8^ In addition, the Americans with Disabilities Act (ADA) as amended specifically counts people with conditions that are episodic, mitigated, or in remission as having a disability.^9^ This inclusive framework contrasts with the ACS-6 questions, which require the current presence of a functional limitation for a person to be counted as having a disability. A more inclusive approach to measurement is consistent with various federal statutory definitions of disability, which recognize the broad and diverse nature of disability.^10^

ACS disability data are used in making decisions about federal, state, and local funding allocations; documenting health disparities; assessing compliance with the ADA; planning for emergencies; and for a host of other governmental and research activities. Clearly, ACS data have real-world implications that affect millions of disabled Americans. The incongruity between disability measurement via the ACS-6 questions vs the broader definitions found in disability and health legislation could lead to underfunding and inadequate support for significant portions of the disability population. Therefore, aligning disability measurement with broader federal conceptualizations is essential for ensuring accurate data collection and equitable resource distribution.^3^

**Figure 1.**
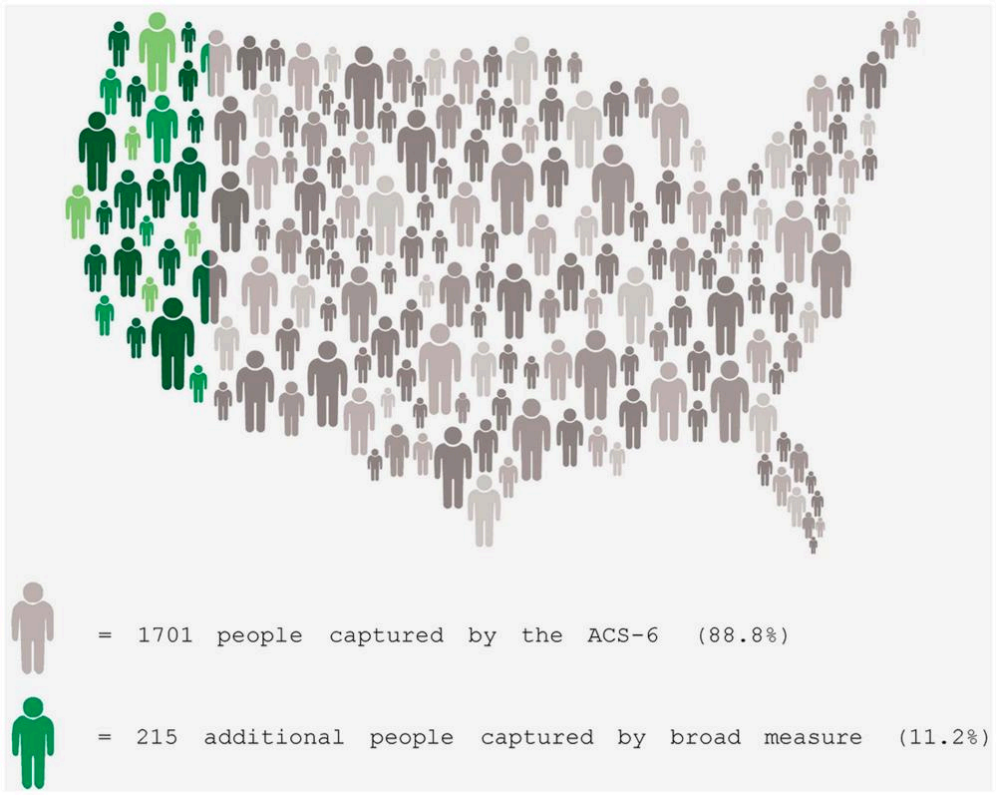
Representation of survey respondents captured by the American Community Survey functional disability questions (ACS-6) and additional respondents only captured by the broad demographic disability question, United States, 2023/2024. Source: Authors’ analysis of data from the 2023/2024 NSHD. Notes: ACS-6 questions include: Are you blind or do you have serious difficulty seeing even when wearing glasses?; Are you deaf or do you have serious difficulty hearing?; Because of a physical, mental or emotional condition, do you have serious difficulty concentrating, remembering or making decisions?; Do you have serious difficulty walking, or climbing stairs?; Do you have difficulty bathing or dressing?; Because of a physical, mental, or emotional condition, do you have difficulty doing errands alone, such as visiting a doctor’s office or shopping? Broad demographic disability question: Do you have any type of health condition, mental health condition, or disability that has lasted or is expected to last for 6 months or more?.

Using a national survey, we compared responses to the ACS-6 functional questions and a broad demographic disability question. We found the broad question captured a substantial portion of respondents not captured by the ACS-6 (Figure 1). We also compared the broad question and ACS-6 responses with two additional questions focused on disability identity and disability type.

## Study data and methods

We used data from the fifth wave of the National Survey on Health and Disability (NSHD; *n* = 1916), a national, internet-based survey of adults ages 18 and older, fielded from October 2, 2023, to February 2, 2024. Participants were recruited through more than eighty disability/chronic illness/mental health organizations, conferences, and meetings. The online survey platform was fully accessible to those with disabilities and respondents had the option to complete the survey by telephone or through a proxy.^11^ The University of Kansas Institutional Review Board approved all study consent forms, instruments, and procedures (Study No. 00147878).

Potential respondents were invited to take the full survey if they answered yes to a broad disability question “Do you have any type of health condition, mental health condition, or disability that has lasted or is expected to last for 6 months or more?” AND/OR answered yes to any one of the current ACS six-question set (ACS-6), which asks whether respondents have difficulty hearing; seeing; thinking, remembering, concentrating, or making decisions; walking/climbing stairs; bathing/dressing; and doing errands alone (Figure 1).

Those screening into the survey via the above questions were asked two additional disability-related questions. Respondents were asked “Do you identify as a person with a disability?” with “yes,” “no,” “prefer not to answer,” or “I don’t know” response options. They were also asked to self-categorize their primary disability type using one of seven categories: “Of the options listed below, which ONE category would you use to describe your main disability or health condition? Intellectual/cognitive, Mental illness/Psychiatric, Physical/Mobility disability, Chronic illness or disease, Sensory, Developmental, Neurological.”

We used SPSS version 29 to conduct descriptive statistics to explore responses to the various disability questions and document which disability groups were most likely to be included or missed by each.

## Study results

Out of the total sample of 1916 respondents, the ACS-6 questions captured 1701 respondents (88.8%). The broad disability question captured 1867 respondents (97.4%), which includes 215 respondents (11.2%) not captured by the ACS-6 questions. Only 49 respondents (2.6%) were identified exclusively by the ACS-6 questions (Figure 1). Of the 1916 respondents screened into the survey, 1734 answered “yes” to the disability identity question (90.5%), 83 answered “no,” and the remaining 99 answered “I don’t know” or “prefer not to answer.”

Next, we examined the types of disabilities captured and missed by the various questions. The ACS-6 questions were significantly more likely (*P* < 0.001) to miss people self-categorizing as having a mental illness (17.8%) or chronic illness (15.5%) and least likely to miss those reporting a sensory disability (0.7%). In contrast, there were no significant differences in the types of disabilities captured or missed by the broad disability question (Table 1), which missed significantly fewer people with psychiatric disabilities (1.7%) compared to the ACS-6 (17.8%).

**Table 1.** Percentage of “no” responses to the ACS-6 and broad disability question within each disability self-categorization, (*n* = 1916) United States, 2023/2024.

Measure	Respondent self-categorized disability type, % (*n*)							
	Intellectual/cognitive	Mental illness/psychiatric	Physical/mobility	Chronic illness or disease	Sensory	Developmental	Neurological	Overall
ACS-6^a^	8.2 (5)	17.8 (41)	6.1 (34)	15.5 (82)	0.7 (1)	12.4 (14)	13.6 (36)	11.3 (213)^b^
Broad question	6.6 (4)	1.7 (4)	2.0 (11)	3.9 (21)	2.2 (3)	2.6 (3)	1.1 (3)	2.6 (49)

Source: Authors’ analysis of data from the 2023/2024 NSHD. Notes: ACS-6 questions: Are you blind or do you have serious difficulty seeing even when wearing glasses?; Are you deaf or do you have serious difficulty hearing?; Because of a physical, mental, or emotional condition, do you have serious difficulty concentrating, remembering, or making decisions?; Do you have serious difficulty walking or climbing stairs?; Do you have difficulty bathing or dressing?; Because of a physical, mental, or emotional condition, do you have difficulty doing errands alone, such as visiting a doctor’s office or shopping?; Broad disability question: Do you have any type of health condition, mental health condition, or disability that has lasted or is expected to last for 6 months or more?; Survey item for disability self-categorization: “Of the options listed below which ONE category would you use to describe your main disability or health condition?” with 7 options randomized.

^a^ *P* < 0.001; chi-square test.

^b^Missing data for self-categorized disability type not included (2 respondents).

The disability identity question also significantly missed respondents with mental illnesses (19.3%), but generally captured slightly larger portions of people in each disability category than did the ACS-6 questions and slightly smaller portions than did the broad screening question.

## Discussion

In 2022, the authors identified shortcomings of the ACS-6 disability question set and noted that some disability types were missed by the ACS-6 more than others.^5^ We advocated for adding a broad, inclusive disability question to federal surveys, as well as an option for people to write in and/or to self-categorize their disability(ies). In 2023, Salinger et al.^6^ noted that 40% of their survey participants responding “yes” to identifying as a person with a disability were missed by the ACS-6 questions, indicating another potential group of people excluded by the current federal disability questions. Importantly, they also found that the sample of people identifying as disabled were more likely to report unfair treatment in health care settings.^6^

In this study, we examined a sample of people who screened into a national survey by answering yes to the existing ACS-6 functional difficulty questions or a broad demographic disability question. We compared responses across these items and looked at types of disabilities missed by each. We found that the single broad disability question captured 11.2% more people than the ACS-6 set alone and did not significantly undercount any disability type. Most respondents (90.5%) screened into the survey also answered yes to the disability identity question.

While this study is limited by a relatively small sample and more testing is needed, these findings provide additional data to support our previous recommendation to add a broad disability question to existing federal survey questions. And, to more fully understand the composition and diversity of the disability population, we strongly suggest adding either a disability self-categorization question or an open-ended question to list disability(ies), similar to questions used for collecting race and ethnicity data in the decennial Census. As the Census Bureau notes, allowing respondents to write-in responses will “more accurately illustrate the richness and complexity of how people identify.”^12^ Given the complexity of capturing disability, such options should absolutely be explored.

Because the new disability questions proposed by the US Census Bureau would have used substantially more space than the existing ACS-6 questions and open-ended, write-in responses for race and ethnicity are already being used, it seems unlikely that the addition of a broad demographic disability question and a categorization or open-ended question to the existing set poses a hardship. In return, the questions would yield a more accurate and inclusive estimate of Americans with disabilities. The more inclusive sample combined with self-categorized or written-in information on disability type would provide more specificity than is currently available. Specificity, in turn, provides a much richer understanding of the disability population, potentially better supporting appropriate program development and funding and allowing for more meaningful analysis of health disparities in this population.

## Supplementary Material

qxae106_Supplementary_Data
